# AnnoTrack - a tracking system for genome annotation

**DOI:** 10.1186/1471-2164-11-538

**Published:** 2010-10-05

**Authors:** Felix Kokocinski, Jennifer Harrow, Tim Hubbard

**Affiliations:** 1 Vertebrate Genome Analysis, Wellcome Trust Sanger Institute, Wellcome Trust Genome Campus, Hinxton, Cambridge, CB10 1HH, UK

## Abstract

**Background:**

As genome sequences are determined for increasing numbers of model organisms, demand has grown for better tools to facilitate unified genome annotation efforts by communities of biologists. Typically this process involves numerous experts from the field and the use of data from dispersed sources as evidence. This kind of collaborative annotation project requires specialized software solutions for efficient data tracking and processing.

**Results:**

As part of the scale-up phase of the ENCODE project (Encyclopedia of DNA Elements), the aim of the GENCODE project is to produce a highly accurate evidence-based reference gene annotation for the human genome. The *AnnoTrack *software system was developed to aid this effort. It integrates data from multiple distributed sources, highlights conflicts and facilitates the quick identification, prioritisation and resolution of problems during the process of genome annotation.

**Conclusions:**

AnnoTrack has been in use for the last year and has proven a very valuable tool for large-scale genome annotation. Designed to interface with standard bioinformatics components, such as DAS servers and Ensembl databases, it is easy to setup and configure for different genome projects. The source code is available at http://annotrack.sanger.ac.uk.

## Background

Even years after the sequencing of the human genome, its annotation is far from complete. The current scale-up phase of the ENCODE project aims to bring us closer to this goal in a collaborative effort between research groups across the world. The output of the GENCODE project within this framework [1], [2] is a set of genes assessed by a number of different methods and considered to be a reference set for other genome analysis. This set includes protein-coding loci with alternative isoforms, non-coding loci with transcript evidence, and pseudogenes. GENCODE includes CCDS project annotation [3] and its output has become the human gene set displayed in Ensembl [4]. Eight groups in Europe and the US directly contribute data to this project along with numerous additional sources of evidence used for the annotation.

Like any large-scale project, the project and data management of GENCODE require substantial effort to allow sufficient progress within the given time frame. It requires specialized software solutions to allow the tracking of different types of annotation and addition of complementary pieces of information over time. Various project management and error tracking programs exist, but the requirements are more specific here:

• Information about genomic localisations needs to be stored in a consistent and searchable way

• The hierarchy between loci, transcripts and exons should be represented

• The different transcripts need to be tracked through a defined process of prediction, annotation and verification

• Multiple heterogeneous data sources have to be integrated

• Various annotation problems should be flagged and then resolved using controlled terms

We could not identify existing software that would allow us to accomplish all these goals. Software in use for similar projects are the Genome Reference Consortium report system [5] and the Mouse Genome Annotation report system [6] which are based on the Jira [7] software. However they haven't been developed to offer an interface rich enough for example to allow the user to quickly find selected types of problems or regions of interest, to use controlled problem descriptions and solutions, and to monitor all past changes. These systems are also not available as open source projects unfortunately. Here we therefore describe the development and application of AnnoTrack, a data tracking system for genome annotation with the hope that similar projects can use and build on it.

## Implementation

AnnoTrack's user interface and underlying database is based on an open-source ticketing system called Redmine [8], a community-organized Ruby on Rails project.

There a number of other good open-source tracking systems available, e.g. RT [9], Bugzilla [10], Trac [11]. The main functionality of tracking issues from projects through a defined flow is the same between all of these. Our choice of Redmine was based on the framework (Ruby on Rails) and the possibility to easily modify major parts of the system.

Most of Redmine's functionality of data tracking, user management and user interface is still in use in AnnoTrack. The following changes and additions have been made with a genome-annotation specific focus. The concept of issues or tickets (describing a certain software problem) belonging to projects (which usually encompass a specific program) has been replaced by the concept of transcripts belonging to a genomic locus. Fields and logic to store genomic locations were added to both levels. As an additional layer "subfeatures" have been introduced to describe coding exons or other parts of transcripts. The option to highlight specific features in the system has been re-introduced as a flexible concept of "flags" (described below).

Where possible changes have been introduced encapsulated in form of a plugin separated from the original Redmine code. A Perl API (application programming interface) has been developed as an abstraction layer on top of the database. This provides an easy and safe way to insert and query tracking data. AnnoTrack's Perl scripts use the higher-level functions provided by this API to integrate heterogeneous data sources and perform data updates and analysis.

## Results

### Data Integration

All GENCODE partners are providing real-time access to their annotation and analysis results via DAS servers (Distributed Annotation System [12]). DAS defines a protocol for client-server communication heavily used in the bioinformatics community [13]. A single client can integrate annotation data from multiple distributed servers on a common coordinate system. The application of DAS to supply genome-wide annotation on this scale is new, however, and poses challenges concerning the number of features, the different annotation formats and the interconnection between them. We addressed these issues by

• Querying the genome in windows of 10 kilobases and filtering out duplications from features overlapping the borders

• Specifying a tight format with a verification web script [14] of the data served by the projects' DAS servers, based of the most recent DAS specifications of the time (1.53E)

• Setting up data parsers and analysis modules for the specific types of annotations (e.g. novel elements to be annotated or elements referring to existing annotation) and data access (a configuration file with definitions of all servers and parameters).

**Figure 1:**
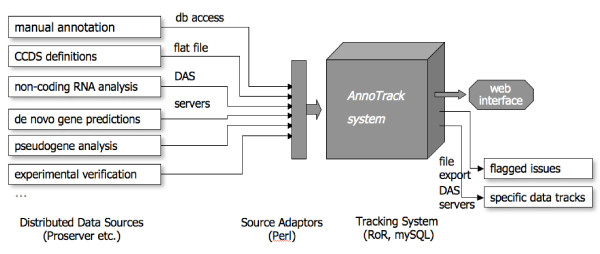
**Layout of the AnnoTrack system**. Input: Heterogeneous sources are accessed by source adaptors and the data is integrated or analysed directly. Output: All data can be retrieved using the Perl API or the web interface; selected data is exported using DAS.

DAS servers and other data sources (flat files, databases) are accessed by the AnnoTrack system using automated Perl scripts in regular intervals; their data is compared to existing annotation and integrated into the database (figure 1). This includes comparing the genomic coordinates as well as the textual descriptions. Specific tags are assigned to the features, indicating the status within the whole process (e.g. "updated", "rejected", "experimentally_verified"). The software identifies disagreements between new and existing data and flags these cases using different levels of priority. Alternatively flags can be set using the web interface or flat files.

### Accessing the Data and Resolving Conflicts

**Figure 2:**
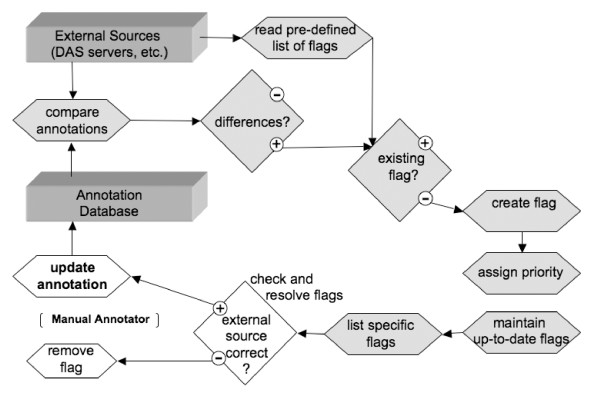
**Flow of data within AnnoTrack**. Annotation comparisons are based on the genomic coordinates. The users are directed to most interesting or most urgent issues to work on. Legend: Rectangles = data sources, diamonds = tests, rhombs = actions, light grey = steps accomplished by the AnnoTrack system.

The typical flow of information to resolve differences in AnnoTrack is outlined in figure 2. The web interface is the main entry point to the system for users. The list of entries can be reviewed and filtered or searched for specific keywords, status information or genomic location. New flags can be set and existing ones can be reviewed. Flags are manually resolved by annotators using controlled vocabulary or automatically resolved if the next data update does not trigger the same conflict. The solution can either be an update of the existing annotation using the new external evidence or a rejection of the external data. Date and user information is automatically stored as a history. Along with general statistics per chromosome or category generated by AnnoTrack, the information on resolved flags is also used to monitor the progress of the entire annotation project itself.

Specific flags can also be set to select transcripts for the experimental verification pipeline (VERIFY) within GENCODE. The list of VERIFY cases and the results can be browsed specifically. High priority transcripts as well as the VERIFY cases are read by one of our DAS servers and can be displayed in the annotation software and other systems. Resolved problems also feed back into the general genome annotation process itself.

### Typical workflow

**Figure 3:**
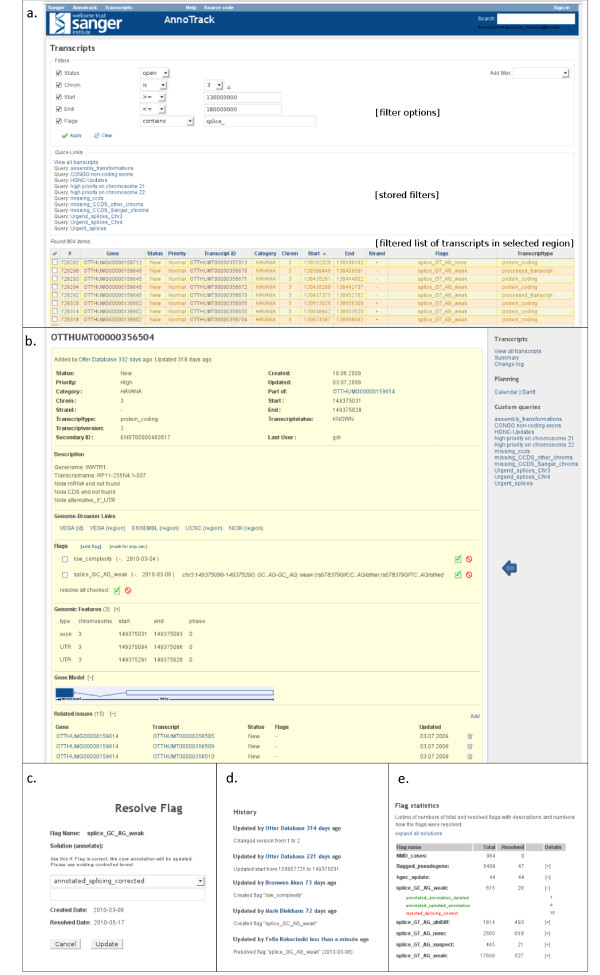
**Example of a transcript under review**. Complete workflow showing the tracking of annotation updates based on an external analysis as described in the text. 3.a: Transcript list page showing filter options, predefined filters and a filtered list of transcripts with open problems in the selected region sorted by genomic start. 3.b: Detail page of a selected transcript from a. with basic annotation data, links to the id or genomic region in public genome browsers, list of flags for this transcripts and links to resolve them individually or combined, coordinates of exons of this transcripts, gene model representation, and links to other transcripts in the region to allow region-wise problem resolution. 3.c: View of page to resolve selected flags from b. with controlled terms. 3.d: History showing all changes. 3.e: Statistics page for monitoring problem solutions. More screenshot are available at [15].

A typical workflow example for an annotator is shown in figure 3 and can be described like the following: Existing manual annotation was analysed with an external computational pipeline by a collaborator. Dubious splice sites were detected and loaded as a defined sets of flags into AnnoTrack using the annotation ids with an AnnoTrack parser module set up to read tab-delimited files. An automated script will pick up the new flags in the database, raise the priorities of transcripts affected and update the statistics. An annotator working on human chromosome 3 can filter the list of AnnoTrack entries to show all "splice" flags in the region (3.a.). Selecting one of the transcripts shows more details (3.b.). The annotator would now view and edit the actual annotation externally and update the transcript's splice sites if he/she agrees with the suggestion. Clicking on the "accept" or "decline" icons on the AnnoTrack transcript page will load a screen showing all controlled terms applicable, e.g. "annotated_splicing_corrected" (figure 3.c). The history of the transcript will display all changes to the transcript and give the annotator the option to add additional comments (3.d.). Summary statistics can be shown to monitor which problems were resolved in what ways (3.e.)

### Using the software

The system is designed to work with any project where there are one or more sets of gene annotations with identifiers and genomic coordinates and other analysis referring to these using either lists of ids or the same coordinate system. Where required, instead of the gene-transcript-exon organisation, all transcript-level entries can be linked to one categorical gene entry and sub-features can be omitted. Links and gene model image sources (currently set up for Ensembl) can be defined in the settings. Additional instructions on how to set up AnnoTrack for a new annotation project are given on the website. Running the system does not require knowledge of the Redmine code or Ruby programming skills, but adjustments to parsers will require knowledge in Perl.

## Conclusions

During the last year AnnoTrack has proven to be a valuable tool to organize the data-intensive task of genome annotation using various data sources and prioritize datasets where manual annotators should re-visit the annotation. More than 4000 issues have been resolved this way in the last year. The information of how the cases were solved helps to improve the data-generating methods and is also used to analyze the progress of the GENCODE project itself. Providing access to the tracking data of some of the manual annotation underlying the GENCODE/Ensembl gene set used here also gives researchers working with these gene sets the chance to quickly learn about known problems or additional information that might be of interest to their research. Feedback from its users is continuing to help improve the application. Future improvements will address better response time (eg. when showing unfiltered lists of all transcripts), automation of additional tasks (e.g. importing external annotation) and creation of further report pages about the progress.

The abstraction and modularity allows the system to be easily adapted and should be of interest to groups working on similar tasks. Besides the main installation described here we have already set up tracking systems internally in a similar way to assist with the annotation of the zebrafish and mouse genomes. The main benefits of this approach are the possibility of

• Highlighting different problems occurring during genome annotation

• Identifying and displaying conflicts within the data along with annotation information at the particular locus

• Providing mechanisms to resolve these conflicts using controlled terms

• Recording a history of all changes and monitoring the overall progress

• Enabling the analysis of how issues where resolved in order to improve the annotation and the external analysis at the same time

The Perl API is documented, data and all source code is available as open source software on our website along with general documentation and screen shots.

## Availability and requirements

- **Project name: **AnnoTrack.

- **Project home page: **http://annotrack.sanger.ac.uk

- **Operating system: **independent, tested on Unix and Mac OS X

- **Programming language: **Ruby (1.8.6), Perl (5.8.1)

- **Other requirements: **MySQL database (5); Rails (2.0.2), BioPerl (1.2.1), EnsEMBL Perl API (currently 58). Please refer to website for full listing.

- **License: **Open Source GNU GPL 2

- **Any restrictions to use by non-academics: **none

## Authors' contributions

FK designed and implemented the AnnoTrack system, JH and TH conceived and advised, all authors read and approved the final manuscript.
